# Association between the nutritional risk index and postmenopausal osteoporosis in patients with type 2 diabetes mellitus

**DOI:** 10.3389/fnut.2026.1801604

**Published:** 2026-04-20

**Authors:** Abuduwupuer Haibier, Hang Lin, Wei Liu, Wencui Li

**Affiliations:** 1 Xinjiang Medical University, Urumqi, Xinjiang Uygur Autonomous Region, China; 2 Sixth Affiliated Hospital of Xinjiang Medical University, Urumqi, China; 3 Shenzhen Second People’s Hospital, Shenzhen, Guangdong, China

**Keywords:** geriatric nutritional risk index, postmenopausal osteoporosis, predictive value, risk factors, type 2 diabetes mellitus

## Abstract

**Objective:**

To investigate the association between the Geriatric Nutritional Risk Index (GNRI) and the presence of postmenopausal osteoporosis (PMOP) in elderly female patients with type 2 diabetes mellitus (T2DM), and to evaluate the discriminatory ability of GNRI for PMOP in this population.

**Methods:**

A retrospective observational study was conducted, enrolling 324 postmenopausal female patients with T2DM who were hospitalized at our hospital from September 2021 to November 2024. Participants were divided into an osteoporosis group (T-score ≤ − 2.5, *n* = 141) and a non-osteoporosis group (T-score > − 2.5, *n* = 183) based on lumbar spine bone mineral density (BMD) measured by dual-energy X-ray absorptiometry. Data on age, body mass index (BMI), BMD, serological indicators, and GNRI were collected and compared between the two groups. Correlation analysis was performed to examine the relationship between GNRI and various parameters. Binary logistic regression was used to identify independent factors influencing PMOP. The predictive efficacy of GNRI was assessed using the receiver operating characteristic (ROC) curve.

**Results:**

Compared to the non-osteoporosis group, patients in the osteoporosis group were significantly older and had significantly lower levels of GNRI, BMI, lumbar spine T-score, total protein, albumin, uric acid, albumin-corrected calcium, serum phosphorus, and 25-hydroxyvitamin D (all *p* < 0.05). Correlation analysis revealed that GNRI was negatively correlated with age (rs = −0.203, *p* < 0.001) and positively correlated with lumbar spine T-score (rs = 0.485, *p* < 0.001), albumin-corrected calcium (rs = 0.532, *p* < 0.001), and 25-hydroxyvitamin D (rs = 0.528, *p* < 0.001). Multivariate logistic regression analysis identified age as an independent risk factor for PMOP (OR = 1.092, 95% CI: 1.038–1.149, *p* = 0.001), while GNRI was independently associated with a lower risk of PMOP (OR = 0.812, 95% CI: 0.680–0.969, *p* = 0.021). ROC curve analysis demonstrated that the area under the curve (AUC) for GNRI in discriminating PMOP was 0.769 (95% CI: 0.718–0.820, *p* < 0.001). The optimal cut-off value was 101.2, with a sensitivity of 70.2% and a specificity of 73.8%.

**Conclusion:**

In postmenopausal female patients with T2DM, a higher GNRI value is independently associated with a lower risk of PMOP. GNRI demonstrates moderate discriminatory ability for identifying PMOP in this population and may serve as a simple and useful auxiliary indicator for assessing skeletal health risk in clinical practice, pending further prospective validation.

## 1 Introduction

Type 2 diabetes mellitus (T2DM) and osteoporosis (OP) are two common chronic diseases that pose significant threats to the health of postmenopausal women. However, the relationship between them is complex. Contrary to the traditional view that patients with T2DM have higher bone mineral density (BMD), a growing body of evidence indicates that their fracture risk is paradoxically increased (1–3). This “diabetic bone paradox” suggests that T2DM affects bone quality through mechanisms beyond BMD, including impaired microarchitecture and abnormal bone turnover, making it a distinct condition termed “diabetes osteoporosis” by some investigators (4). Consequently, identifying modifiable risk factors for osteoporosis in this specific population is of paramount clinical importance.

In patients with T2DM, declining renal function represents a critical link between metabolic dysregulation and bone fragility. As estimated glomerular filtration rate (eGFR) decreases, the 1α-hydroxylation of 25-hydroxyvitamin D in the kidney is progressively impaired, leading to reduced circulating levels of active 1,25-dihydroxyvitamin D (5). This, in turn, diminishes intestinal calcium absorption and contributes to secondary hyperparathyroidism, a state characterized by increased osteoclastic bone resorption and accelerated bone loss (6). Moreover, chronic kidney disease—commonly encountered in long-standing T2DM—is frequently accompanied by metabolic acidosis, even in its mild, subclinical form. Under such conditions, the body mobilizes alkaline salts from bone to buffer excess hydrogen ions, resulting in the release of calcium and phosphate from the mineralized matrix and promoting osteoclast activity (7). Over time, this acid-induced bone resorption can further compromise bone mineral density and skeletal microarchitecture. Collectively, these interconnected pathways underscore the importance of considering both renal function and acid–base status in evaluating skeletal health in patients with T2DM.

Among the myriad factors affecting bone health in T2DM, nutritional status has emerged as a fundamental and particularly critical modifiable dimension (4). Patients with diabetes often face heightened nutritional risks due to strict dietary management, diabetes-related complications (e.g., gastroparesis, nephropathy), and the catabolic effects of chronic hyperglycemia. This nutritional vulnerability is not limited to protein-energy malnutrition but also includes deficiencies in micronutrients essential for bone metabolism, such as calcium and vitamin D (5). Importantly, the pathophysiology of diabetic osteoporosis is now understood to involve not just hyperglycemia, but also sarcopenia, chronic low-grade inflammation, and gut microbiota dysbiosis—all of which are closely intertwined with nutritional status (4). Therefore, a comprehensive assessment of nutritional risk may offer a crucial window into understanding and managing skeletal fragility in this population.

Despite this recognized importance, current research largely focuses on the associations of single nutrients or isolated biomarkers with BMD in diabetic patients. There is a notable gap in studies utilizing a comprehensive, systematic nutritional risk assessment to evaluate its relationship with osteoporosis, particularly in the high-risk subgroup of postmenopausal women with T2DM (8). This population faces a convergence of risk factors—aging, estrogen deficiency, and the metabolic challenges of diabetes—making the integrated assessment of nutritional status a potentially powerful tool for risk stratification. Given this gap, a retrospective study design offers an efficient approach to explore these associations using existing clinical data (9).

This study, designed as a retrospective observational analysis, aims to quantitatively evaluate the association between comprehensive nutritional risk status and the risk of osteoporosis in postmenopausal T2DM patients through systematic analysis of historical clinical data. The study seeks to delineate the distribution profile of the Geriatric Nutritional Risk Index (GNRI) within this specific population and, employing multivariate logistic regression models while controlling for known confounders, to test whether GNRI serves as an independent predictor of osteoporosis. Its core objective is to move beyond single nutritional indicators, providing new epidemiological evidence from the perspective of overall nutritional status to understand the pathophysiological mechanisms of diabetic bone disease.

## 2 Subjects and methods

### 2.1 Patient selection

This was a single-center, retrospective, observational study. Data were derived from postmenopausal female patients (aged ≥60 years) with T2DM who were hospitalized at our hospital between September 2021 and November 2024. Based on strict inclusion and exclusion criteria, a total of 324 patients were ultimately enrolled in the analysis. The study protocol was approved by the Institutional Review Board of The Sixth Affiliated Hospital of Xinjiang Medical University. As a retrospective analysis utilizing only anonymized existing clinical data without imposing additional risks, the ethics committee granted a waiver of individual informed consent.

### 2.2 Inclusion criteria

The inclusion criteria were as follows: (1) diagnosis of T2DM based on established criteria; (2) age ≥60 years; (3) postmenopausal status; and (4) availability of complete records for height and weight measurements, dual-energy X-ray absorptiometry (DXA) scans for BMD, and required blood biochemical and bone metabolism marker tests during the hospitalization.

### 2.3 Exclusion criteria

Patients were excluded if they met any of the following criteria: (1) comorbid conditions known to affect bone metabolism or nutritional status, such as malignancies or severe hepatic or renal insufficiency; (2) diagnosis of diabetes mellitus other than T2DM (e.g., type 1 diabetes); (3) presence of thyroid or parathyroid disorders; (4) long-term use of medications affecting bone metabolism (e.g., glucocorticoids); (5) severe psychological or cognitive impairment precluding adequate cooperation; or (6) incomplete clinical data.

### 2.4 Diagnostic criteria

#### 2.4.1 Diagnostic criteria for osteoporosis

The diagnosis of OP was established according to the 2022 Chinese guidelines for the diagnosis and treatment of primary osteoporosis (10). In addition, we referred to the Chinese expert consensus on the management of fracture risk in patients with diabetes, which was jointly issued by the Chinese Society of Endocrinology and three other national societies (11). According to these guidelines, lumbar spine BMD was measured using dual-energy X-ray absorptiometry (DXA). A T-score ≥ − 1.0 was classified as normal bone mass, a T-score between −2.5 and −1.0 as osteopenia, and a T-score ≤ − 2.5 as osteoporosis. While it is recognized that this T-score threshold may underestimate fracture risk in diabetic patients, it remains the current practical diagnostic criterion for initiating anti-osteoporosis therapy in clinical practice in China, as recommended by the consensus (11).

#### 2.4.2 Diagnostic criteria for T2DM

The diagnosis of T2DM was based on the criteria outlined in the Chinese guidelines for the prevention and treatment of type 2 diabetes (2020 edition) (12). Furthermore, type 1 diabetes and other specific types of diabetes were rigorously excluded. Features leading to exclusion included: age of onset <30 years, presentation with classic symptoms of polydipsia, polyphagia, polyuria, and weight loss or with ketoacidosis at onset, non-obese body type, significantly reduced C-peptide levels, or the presence of insulin autoantibodies.

### 2.5 Clinical data collection

Clinical data were collected retrospectively through the hospital information system. Data included:

1) Basic information: age.
2) Anthropometric measurements: Height (cm) and weight (kg) were measured by nurses with patients in a fasting state, wearing light clothing and without shoes. BMI was calculated as weight (kg) divided by height squared (m^2^).
3) Laboratory parameters: Fasting venous blood samples were collected after an 8-h fast for analysis. Measured parameters included serum albumin (Alb), creatinine (Scr), uric acid (UA), calcium (Ca), phosphorus (P), magnesium (Mg), triglycerides (TG), total cholesterol (TC), 25-hydroxyvitamin D [25(OH)D], glycated hemoglobin (HbA1c), eGFR, hemoglobin (Hb), prealbumin (PA), and the bone turnover markers *β*-C-terminal telopeptide of type I collagen (β-CTx) and procollagen type I N-terminal propeptide (PINP). To account for the influence of albumin levels on calcium measurements, the albumin-corrected calcium was calculated using the following standard formula: Corrected calcium (mmol/L) = total calcium (mmol/L) + 0.02 × [40 – serum albumin (g/L)].

a) Bone Mineral Density Measurement: Lumbar spine (L1-L4) bone mineral density (g/cm^2^) and corresponding T-scores were measured by certified radiology technicians using a DXA scanner.
b) Calculation of GNRI:

GNRI was calculated using the following formula (13):

GNRI = 1.489 × serum albumin (g/L) + 41.7 × (current weight/ideal weight).

Ideal body weight for women was calculated as: Height (cm)—100—[(Height (cm)—150)/2.5].

If the patient’s current weight exceeded the calculated ideal weight, the ratio (current weight / ideal weight) was set to 1 for the calculation.

Based on the original study by Bouillanne et al. (13), a GNRI value ≤ 98 was defined as indicating nutritional risk, while a GNRI value > 98 was defined as no nutritional risk. This cutoff has been widely validated in elderly and chronically ill populations, including patients with type 2 diabetes (8, 13).

### 2.6 Statistical analysis

Statistical analyses were performed using SPSS software (version 26.0). Continuous data conforming to a normal distribution were presented as mean ± standard deviation (*x-±s*) and compared between groups using the independent samples t-test. Non-normally distributed continuous data were presented as median with interquartile range [*M (P25, P75)*] and compared using the Mann–Whitney U test. Categorical data were expressed as numbers with percentages [*n* (%)] and compared using the Chi-square test. The correlation between GNRI and various clinical parameters was examined using Pearson’s correlation analysis for normally distributed data and Spearman’s rank correlation analysis for non-normally distributed data. Binary logistic regression analysis was employed to identify independent risk factors for postmenopausal osteoporosis (PMOP). The predictive performance of GNRI for PMOP was evaluated using Receiver Operating Characteristic (ROC) curve analysis, and the area under the curve (AUC) was calculated. A two-tailed *p*-value of less than 0.05 was considered statistically significant. Variables with a *p*-value < 0.05 in univariate analysis, along with GNRI, were entered into the multivariate model using a forward stepwise method; variables that did not differ significantly between groups in univariate analysis were not included to maintain model parsimony.

## 3 Results

### 3.1 Participant flow analysis

A total of 324 postmenopausal female patients with T2DM who met the eligibility criteria were enrolled in this study. Based on the BMD T-scores determined by DXA, participants were categorized into two groups: the non-osteoporosis group (T-score > − 2.5; *n* = 183) and the osteoporosis group (T-score ≤ − 2.5; *n* = 141). Complete clinical data were available for all enrolled cases, and all were included in the final statistical analysis without any loss to follow-up or data exclusion.

### 3.2 Grouping flowchart

**Figure 1:**
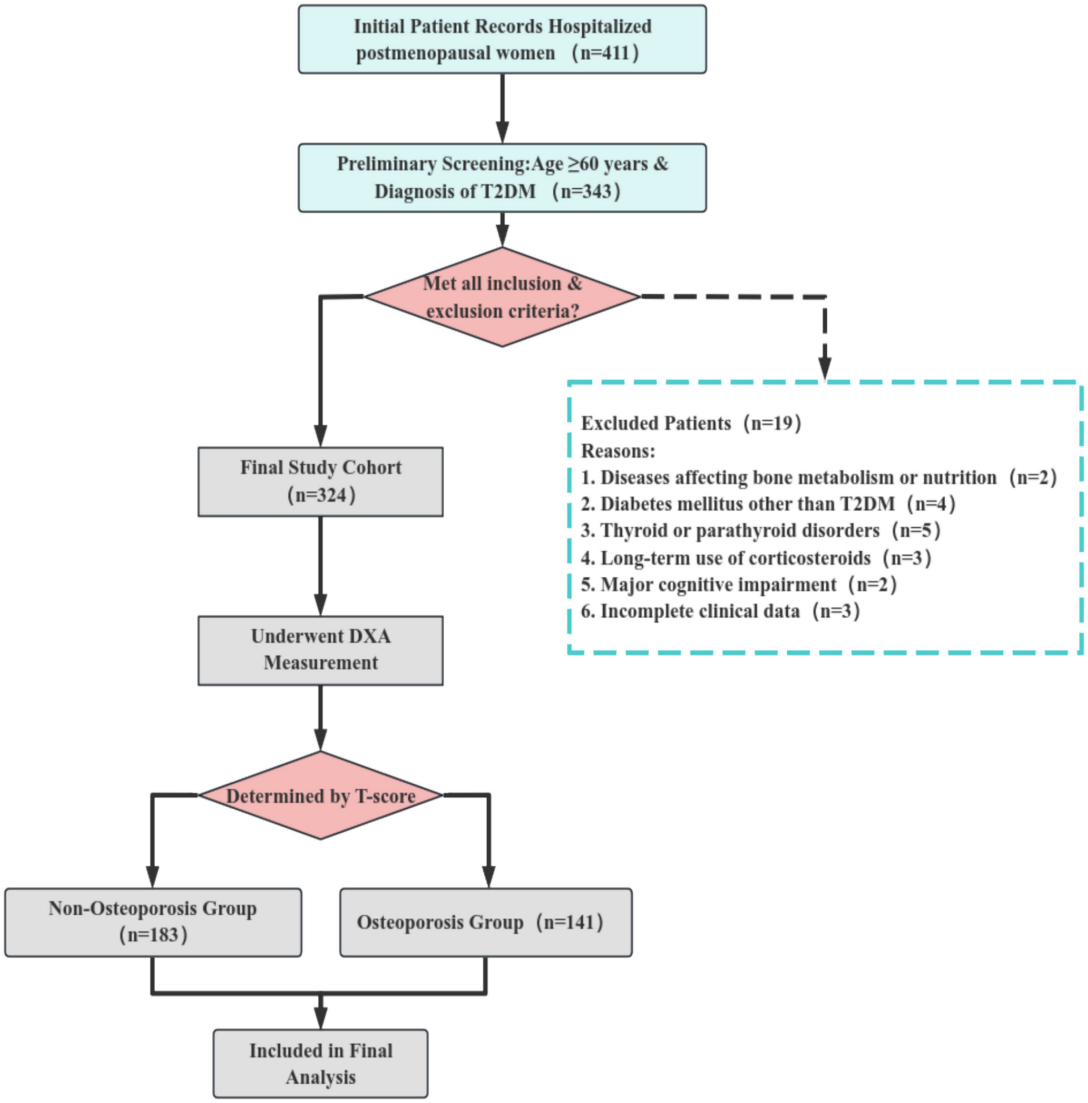
Flow chart of patient assignment.

The flowchart detailing the patient selection and group assignment process is presented in Figure 1.

### 3.3 Comparison of clinical indicators between different bone density groups

As shown in Table 1, compared to the non-osteoporosis group, patients in the osteoporosis group were significantly older (*Z* = −5.236, *p* < 0.001). Meanwhile, their BMI (*t* = 2.854, *p* = 0.005), lumbar spine BMD (*Z* = −9.147, *p* < 0.001), serum total protein (*t* = 7.432, *p* < 0.001), albumin (*t* = 9.226, *p* < 0.001), uric acid (*Z* = −5.021, *p* < 0.001), serum calcium (*Z* = −4.388, *p* < 0.001), serum phosphorus (*t* = 3.031, *p* = 0.003), 25(OH)D (*Z* = −3.890, *p* < 0.001), and GNRI (*Z* = −8.530, *p* < 0.001) were all significantly lower, with the differences being statistically significant. No statistically significant differences were observed between the two groups in serum creatinine, eGFR, magnesium, TG, or TC levels (*p* > 0.05).

**Table 1:** Comparison of baseline characteristics between different bone mineral density groups.

Variable	Non-osteoporosis group (*n* = 183)	Osteoporosis group (*n* = 141)	Statistic	*P*
Age (years)	66.0 (63.0, 71.0)	71.0 (67.0, 75.0)	*Z* = −5.236	<0.001
BMI (kg/m^2^)	24.8 ± 3.4	23.5 ± 4.0	*t* = 2.854	0.005
Lumbar spine T-score	−1.2 (−2.0, −0.2)	−2.9 (−3.6, −2.6)	Z = −9.512	<0.001
Total protein (g/L)	71.2 ± 6.2	65.8 ± 6.5	*t* = 7.432	<0.001
Albumin (g/L)	42.1 ± 4.2	37.8 ± 3.9	*t* = 9.226	<0.001
Serum creatinine (μmol/L)	59.0 (50.0, 69.0)	58.0 (49.5, 68.0)	*Z* = −0.658	0.511
eGFR (mL/min/1.73m^2^)	78.0 (68.0, 89.0)	76.0 (66.0, 87.0)	Z = −1.023	0.306
Uric acid (μmol/L)	330.0 (270.0, 388.0)	268.0 (225.0, 340.0)	*Z* = −5.021	<0.001
Albumin-corrected calcium (mmol/L)	2.31 (2.23, 2.39)	2.24 (2.14, 2.32)	*Z* = −4.412	<0.001
Serum phosphorus (mmol/L)	1.17 ± 0.18	1.11 ± 0.17	*t* = 3.031	0.003
Serum magnesium (mmol/L)	0.88 (0.82, 0.94)	0.86 (0.79, 0.92)	*Z* = −1.706	0.088
25(OH)D (ng/mL)	16.8 (13.5, 20.1)	14.9 (11.8, 18.0)	*Z* = −3.890	<0.001
TG (mmol/L)	1.72 (1.25, 2.42)	1.61 (1.10, 2.28)	*Z* = −1.595	0.111
TC (mmol/L)	4.81 ± 1.02	4.75 ± 1.05	*t* = 0.524	0.601
GNRI	103.8 (99.1, 108.5)	96.5 (92.0, 101.1)	*Z* = −8.530	<0.001

Data are presented as mean ± standard deviation (x̅±s) for normally distributed variables, compared by independent samples *t*-test; or as median (interquartile range) [M (P25, P75)] for non-normally distributed variables, compared by Mann–Whitney *U* test. BMI, Body Mass Index; 25(OH)D, 25-hydroxyvitamin D; GNRI, Geriatric Nutritional Risk Index. Significant *p* values (<0.05) are highlighted in bold.

### 3.4 Comparison of clinical indicators between different nutritional risk groups

In a secondary analysis, patients were stratified according to their GNRI values using a cutoff of 98 (10). Patients with nutritional risk (GNRI ≤ 98, *n* = 115) were significantly older, and had significantly lower BMI, lumbar spine BMD, T-score, 25(OH)D, total protein, uric acid, albumin-corrected calcium, phosphorus, magnesium, and triglycerides compared to those without nutritional risk (GNRI > 98, *n* = 209) (all *p* < 0.05, data not shown). These findings further support the association between GNRI and bone-related parameters.

### 3.5 Correlation analysis between GNRI and clinical indicators

As shown in Table 2, Pearson/Spearman correlation analysis revealed that the GNRI was negatively correlated with age (rs = −0.203, *p* < 0.001). In contrast, GNRI showed positive correlations with BMI (*r* = 0.172, *p* = 0.002), lumbar spine T-score (rs = 0.485, *p* < 0.001), 25(OH)D (rs = 0.218, *p* < 0.001), serum total protein (*r* = 0.728, *p* < 0.001), uric acid (rs = 0.225, *p* < 0.001), serum calcium (rs = 0.532, *p* < 0.001), serum phosphorus (*r* = 0.165, *p* = 0.003), serum magnesium (rs = 0.168, *p* = 0.002), TG (rs = 0.161, *p* = 0.004), and TC (*r* = 0.148, *p* = 0.008). No significant correlation was found between GNRI and serum creatinine (*p* > 0.05).

**Table 2:** Correlation analysis between GNRI and clinical indicators.

Indicator	Correlation coefficient (*r*/rs)	*p*
Age	−0.203	<0.001
BMI	0.172	0.002
T-score	0.485	<0.001
25(OH)D	0.218	<0.001
Total protein	0.728	<0.001
Serum creatinine	−0.010	0.854
Uric acid	0.225	<0.001
Albumin-corrected calcium	0.528	<0.001
Serum phosphorus	0.165	0.003
Serum magnesium	0.168	0.002
TG	0.161	0.004
TC	0.148	0.008

GNRI, Geriatric Nutritional Risk Index; BMI, Body Mass Index; BMD, Bone Mineral Density; 25(OH)D, 25-hydroxyvitamin D; TG, Triglycerides; TC, Total Cholesterol. “*r*” denotes Pearson’s correlation coefficient; “rs” denotes Spearman’s rank correlation coefficient.

### 3.6 Binary logistic regression analysis of risk factors for postmenopausal osteoporosis

Using the presence or absence of osteoporosis as the dependent variable, variables identified as significant in the univariate analysis along with GNRI were included in a multivariate binary logistic regression analysis (forward stepwise method). The results (Table 3) indicated that age (OR = 1.092, 95% CI: 1.038–1.149, *p* = 0.001) was an independent risk factor for PMOP in postmenopausal female patients with T2DM, while GNRI (OR = 0.812, 95% CI: 0.680–0.969, *p* = 0.021) was independently associated with a lower risk of PMOP.

**Table 3:** Multivariate binary logistic regression analysis of risk factors for postmenopausal osteoporosis.

Variable	*β* coefficient	Standard error	Wald *χ^2^*	*p*	OR value	95% CI
Age	0.088	0.027	10.724	**0.001**	**1.092**	**1.038–1.149**
GNRI	−0.208	0.090	5.321	**0.021**	**0.812**	**0.680–0.969**
Constant	3.245	1.502	4.669	0.031	25.672	–

OR, odds ratio; CI, confidence interval; GNRI, Geriatric Nutritional Risk Index.Bold values represent statistically significant differences.

### 3.7 Discriminatory ability of GNRI for postmenopausal osteoporosis

**Figure 2:**
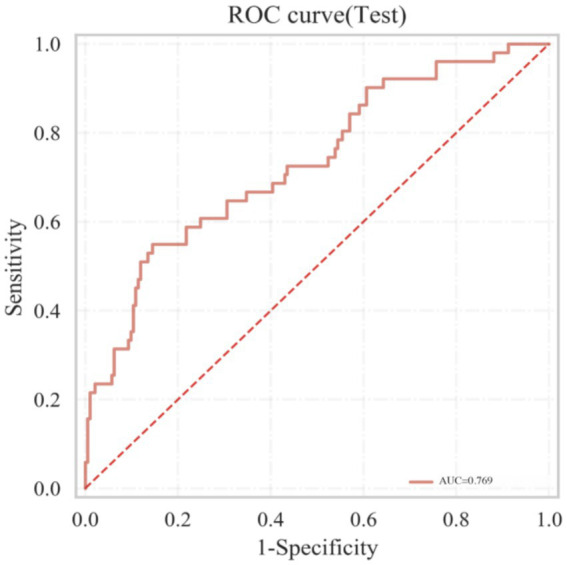
ROC curve of GNRI for discriminating postmenopausal osteoporosis.

ROC curve analysis demonstrated (Figure 2) that the AUC for GNRI in discriminating PMOP in postmenopausal women with T2DM was 0.769 (95% CI: 0.718–0.820, *p* < 0.001), indicating moderate discriminatory ability. The optimal cut-off value was 101.2, as determined by maximizing the Youden index, yielding a sensitivity of 70.2% and a specificity of 73.8% (Youden index = 0.440).

## 4 Discussion

Through a retrospective cohort analysis of 324 postmenopausal female patients with T2DM, this study systematically investigated the association between the GNRI and PMOP, as well as its discriminatory ability. The core findings reveal that GNRI values were significantly lower in the osteoporosis group compared to the non-osteoporosis group. Furthermore, multivariate analysis identified GNRI as independently associated with a lower risk of PMOP, demonstrating moderate discriminatory ability for PMOP with an AUC of 0.769. This finding holds significant importance within the context of the existing literature. Previous research on diabetes and bone health has predominantly focused on the effects of hyperglycemia, insulin resistance, accumulation of advanced glycation end products (AGEs), or specific antidiabetic medications. Our study expands the perspective to the fundamental and modifiable dimension of systemic nutritional status (14, 15). These findings align with the original concept proposed by Bouillanne et al., who introduced GNRI to assess the link between nutritional risk and clinical outcomes in elderly patients, and further specify its application to the complex field of diabetic bone disease (13). GNRI integrates measures of protein reserve (via serum albumin) and energy balance (via body weight status), which correspond to the two pillars of maintaining skeletal health: adequate protein supply is essential for sufficient bone matrix synthesis, while appropriate body weight provides the necessary mechanical load for bone mass preservation (16–18). The positive correlations observed in this study between GNRI and indicators such as lumbar spine BMD, serum calcium, and 25-hydroxyvitamin D suggest that nutritional risk may act as a critical link connecting diabetic metabolic dysregulation to increased bone fragility, potentially by disrupting the homeostasis of key substances involved in bone mineralization and affecting the skeletal structure itself.

This study found that GNRI values were significantly lower in patients in the osteoporosis group compared to the non-osteoporosis group, which is consistent with observations from multiple studies in elderly or chronically ill populations. As a composite index, GNRI encompasses both serum albumin (reflecting protein nutritional status) and body weight status (reflecting energy balance) (19). This study further revealed that GNRI showed strong positive correlations with skeletal indicators such as lumbar spine T-score (rs = 0.485, *p* < 0.001), and was also significantly correlated with levels of key nutrients involved in bone metabolism, including albumin-corrected calcium and 25(OH)D. This suggests that the “nutritional risk” represented by GNRI is not a single deficiency, but rather a state of systemic malnutrition closely associated with bone health. It is noteworthy that, although T2DM patients are traditionally associated with obesity, the osteoporosis group in this study had a significantly lower BMI, and GNRI was positively correlated with BMI (20). This highlights the importance of emphasizing both “quality and weight management” in diabetes care—the need to control blood glucose while also preventing skeletal damage resulting from excessively low body weight and malnutrition (21, 22). A study by Am et al. found that approximately half of the female OP patients had vitamin D levels below the normal range. Since 25(OH)D is the key hormone regulating intestinal calcium absorption, its deficiency directly impedes normal calcium uptake, leading to calcium imbalance in the body (23).

Multivariate logistic regression confirmed that GNRI remained independently associated with a lower risk of PMOP after adjusting for confounders such as age. The optimal cut-off value of 101.2, identified through ROC curve analysis, provides an objective and quantifiable threshold for identifying individuals at potential risk. With a sensitivity of approximately 70% and a specificity of around 74%, GNRI demonstrates potential utility as a low-cost, easily accessible indicator for risk stratification. However, these findings should be interpreted with caution given the cross-sectional design of the study, and the clinical utility of GNRI requires further validation in prospective cohorts. Although it cannot replace DXA as the diagnostic gold standard, this index may assist in identifying postmenopausal T2DM patients with high nutritional risk who could be prioritized for further bone health evaluation. However, the clinical utility of GNRI for guiding interventions requires validation in prospective studies. This suggests a potential role for GNRI in integrating nutritional assessment into skeletal health risk stratification (8, 17).

The association between GNRI and PMOP may be mediated through multiple biological pathways (24). Firstly, hypoalbuminemia may directly impair bone matrix synthesis and affect the transport of vitamin D and its binding proteins, indirectly leading to disturbances in calcium and phosphorus metabolism and secondary hyperparathyroidism (6, 25). Secondly, low body weight and concomitant muscle loss reduce the mechanical load on bones, weakening the key mechanical stimuli necessary for maintaining bone mass (26, 27). Additionally, malnutrition is often accompanied by a state of chronic low-grade inflammation, where elevated inflammatory cytokines (such as TNF-*α* and IL-6) can activate signaling pathways such as nuclear factor kappa B (NF-κB), promoting osteoclastogenesis and inhibiting osteogenesis (26, 28). Therefore, a decline in GNRI can be regarded as a comprehensive composite indicator, signaling that multiple adverse skeletal factors including protein-energy malnutrition, increased inflammatory burden, and diminished mechanical stimulation may be acting in concert to exacerbate skeletal fragility (29, 30).

Emerging evidence has highlighted the potential impact of glucose-lowering therapies on *muscle mass*, which may in turn influence bone health in patients with type 2 diabetes (31). Glucagon-like peptide-1 receptor agonists (GLP-1 RAs), while highly effective for glycemic control and weight reduction, have been associated with variable degrees of lean body mass loss, with some studies reporting that 40%–60% of total weight loss may comprise lean mass (32). Although the clinical significance of this phenomenon remains debated, it is particularly relevant in older adults and those with pre-existing sarcopenia, who may be at greater risk of functional decline and fractures (33). Importantly, sarcopenia and osteoporosis frequently co-exist as “osteosarcopenia,” a condition associated with higher fracture risk and worse clinical outcomes. In this context, nutritional status—as captured by GNRI—may serve as a critical link, because inadequate protein intake and energy deficiency not only contribute to bone loss but also exacerbate muscle wasting. Therefore, in patients receiving GLP-1 RAs or other weight-lowering therapies, regular assessment of nutritional risk using tools such as GNRI could help identify individuals who may benefit from targeted nutritional support and resistance exercise interventions to preserve both muscle and bone mass. Further prospective studies are needed to explore whether nutritional optimization can mitigate therapy-induced lean mass loss and its skeletal consequences.

The conclusions of this study should be interpreted with consideration of the following limitations. First, as a single-center retrospective investigation, it is inherently susceptible to selection bias, and the design precludes the inference of a causal relationship between GNRI and PMOP; thus, the findings necessitate validation through prospective studies. Second, the cohort consisted solely of hospitalized patients, who likely present with greater disease severity and higher nutritional risk compared to the general community population. This may limit the generalizability of our results. Third, despite adjusting for several known confounders, important variables potentially influencing bone metabolism such as precise postmenopausal duration, detailed dietary calcium intake, habitual physical activity levels, and the specific use of certain glucose-lowering medications were not fully accounted for, potentially leading to residual confounding. eGFR was collected and showed no significant difference between the two groups (*p* > 0.05); therefore, it was not entered into the multivariate logistic regression model. Nevertheless, given the well-established role of renal function in bone metabolism, we acknowledge that residual confounding related to renal function cannot be completely excluded, and future studies with larger sample sizes and more detailed renal function stratification are warranted. Fourth, parathyroid hormone (PTH) levels were not measured in this study, as they were not part of the routine clinical protocol during the study period. Given the well-recognized role of secondary hyperparathyroidism in diabetic bone disease—particularly in the context of vitamin D deficiency and potential renal impairment—the absence of PTH data limits our ability to fully characterize the mechanisms underlying the observed associations. Fifth, acid–base balance parameters (e.g., serum bicarbonate) were not available in this study. Chronic metabolic acidosis, which may develop in T2DM patients with declining renal function or suboptimal glycemic control, is known to promote bone resorption through buffering mechanisms that mobilize calcium and phosphate from the bone matrix. The absence of these data precludes assessment of this potential confounder and represents an additional limitation in interpreting the mechanistic pathways linking nutritional status to bone health. Sixth, the study did not incorporate a broader panel of bone turnover biomarkers or advanced imaging modalities to assess bone microstructure. Consequently, a deeper exploration of the underlying mechanisms from the perspectives of dynamic bone remodeling equilibrium and bone quality was not feasible.

## 5 Conclusion

In summary, among postmenopausal women with T2DM, a higher GNRI value is independently associated with a lower risk of osteoporosis. A lower GNRI level is significantly associated with an increased risk of osteoporosis and demonstrates moderate discriminatory ability for identifying this condition in the study population. These findings suggest that GNRI may serve as a simple and useful auxiliary indicator for assessing skeletal health risk in postmenopausal T2DM patients. For individuals with low GNRI values, enhanced nutritional evaluation and bone mineral density monitoring should be considered to facilitate early prevention and management of osteoporosis and related fractures. Further prospective studies are warranted to validate the clinical utility of GNRI in this population.

## Data Availability

The raw data supporting the conclusions of this article will be made available by the authors, without undue reservation.
